# Single-Cell RNA-seq Analysis Reveals Dysregulated Cell-Cell Interactions in a Tumor Microenvironment Related to HCC Development

**DOI:** 10.1155/2022/4971621

**Published:** 2022-05-18

**Authors:** Zhenhao Liu, Siwen Zhang, Jian Ouyang, Dan Wu, Lanming Chen, Wen Zhou, Lu Xie

**Affiliations:** ^1^Department of Hematology, Xiangya Hospital, Central South University, Changsha, Hunan, China; ^2^Key Laboratory of Carcinogenesis and Cancer Invasion, Ministry of Education; Key Laboratory of Carcinogenesis, National Health and Family Planning Commission; Cancer Research Institute, School of Basic Medical Science, Central South University, Changsha, Hunan, China; ^3^Shanghai-MOST Key Laboratory of Health and Disease Genomics, Institute for Genome and Bioinformatics, Shanghai Institute for Biomedical and Pharmaceutical Technologies, Shanghai, China; ^4^Key Laboratory of Quality and Safety Risk Assessment for Aquatic Products on Storage and Preservation (Shanghai), China Ministry of Agriculture, College of Food Science and Technology, Shanghai Ocean University, Shanghai, China 201306; ^5^College of Food Science and Technology, Shanghai Ocean University, Shanghai, China 201306; ^6^Center for Biomedical Informatics, Shanghai Children's Hospital, Shanghai Jiao Tong University, Shanghai, China 200040; ^7^Bioinformatics Center, National Clinical Research Centre for Geriatric Disorders, Department of Geriatrics, Xiangya Hospital, Central South University, Changsha, 410008 Hunan, China

## Abstract

The heterogeneity of tumor microenvironment (TME) of hepatocellular carcinoma (HCC) may relate to cell-cell interaction event (CCE) dysregulation and would affect therapeutic responses and clinical outcomes. To reveal the differentiation of CCEs in the liver tissue from healthy donors (HD) to HCC, scRNA-seq data of ~62000 cells from HD, paracancerous nontumor tissue (NT), and HCC were analyzed. The microenvironmental CCE landscape was constructed. Dysregulated cell types and changed molecular functions were identified with CCE alterations in HCC. Dysregulated CCEs which function as pivotal roles in tumorigenesis and development of HCC included SPP1-CD44, MIF-TNFRSF14, and VEGFA-NRP1. A CCE-based immune regulatory network was extracted to illustrate the mechanism of TME dysregulation. A prognostic signature based on CCE genes was identified and validated in independent datasets. Our study provided insights into the characteristics of the cross-talk between tumor cells and microenvironment in HCC and established a workflow strategy for CCE analyses based on scRNA-seq data.

## 1. Introduction

Hepatocellular carcinoma (HCC) is observed worldwide with a high mortality rate [1], accounting for 8.2% of all cancer deaths [2]. Tumor-promoting inflammation in the liver remains a common feature of the pathogenesis of HCC among all etiologies, accompanied by extensive immune infiltration [3, 4]. Currently, HCC is often treated by surgical resection, chemotherapy, drug therapy, and other methods, but with a high recurrence rate [5]. Immunotherapy has become a key strategy and dramatically altered the oncological treatment landscape in the recent decades [4, 6].

The heterogeneity of the tumor microenvironment (TME) and the alteration of the TME may be key players for HCC immunotherapy [7, 8]. The TME shows a composition of tumor tissue consisting of malignant, immune, and stromal cells, defined by spatiotemporal interactions among heterogeneous cell types [9]. From bulk tissue gene expression profiles, computational methods such as CIBERSORT could quantify cell fractions especially tumor-infiltrating leukocytes in TME [10]. While from single-cell transcriptomics data, predicting enriched cellular interactions between cell types in TME has become accessible [11].

Recent single-cell sequencing studies on HCC reveals the pivotal role of TME and its cellular interactions. HCC was found with varying degrees of heterogeneity. T cells from higher heterogeneous tumors showed lower cytolytic activities, associated with patient's worse overall survival [8]. A cluster of LAMP3+ dendritic cells (DCs) was potentially regulating multiple subtypes of lymphocytes in HCC TME by expressed diverse immune relevant ligands [12]. Hepatocyte-derived VEGFA as ligand could activate PLVAP in tumor fetal-liver-associated ECs, which may participate in the regulation of EC fenestration during fetal-liver organogenesis as well as angiogenesis in HCC [13]. Further studies are necessary to dissect the interaction mechanism between malignant cells and surrounding cell types in the HCC TME.

In our previous work, the proportion of plasma cells in the HCC TME was found related to the development from cirrhosis to cancer in patients, suggesting the important role of immunoregulation [14]. However, the causes of these results were poorly defined. In this study, based on scRNA-seq data, the cellular interactions in HCC, paracancerous nontumor tissue, and healthy liver tissue were investigated. The alterations of cell-cell interaction events (CCEs) were identified. Key molecules and functional pathways in HCC caused by the CCE changes, and the related molecular pathogenesis of HCC was further explored. An immune-associated prognostic model was established, which may be proved referable for studying the characteristics and mechanisms of dysregulated tumor microenvironment in hepatocellular carcinoma.

## 2. Material and Methods

### 2.1. scRNA-seq Datasets and Bulk RNA-seq Dataset Collection

To reveal microenvironmental alterations between HCC and healthy liver tissue, single-cell RNA-seq data were collected from Gene Expression Omnibus (GEO). Single-cell transcriptomic data from primary tumor and paired nontumor liver (paracancerous liver tissue) of 5 patients (HCC03, HCC04, HCC05, HCC06, and HCC09, without metastatic) were downloaded from the GEO dataset GSE149614. And single-cell transcriptomic data of healthy livers were downloaded from the GEO dataset GSE136103 [15]. Cellranger, the single-cell software suite from 10X Genomics, was used for alignment and counting analysis with the reference genome (GRCh38). Matrix generated by Cellranger was downloaded. Further, bulk RNA-seq data for samples with HCC and healthy liver tissue (or adjacent normal liver tissue) were downloaded from TCGA LIHC datasets and the GEO dataset GSE14520 [16].

### 2.2. Cell Type Annotation and Malignant Cell Identification with scRNA-seq

The data matrixes were downloaded from GEO and prepared for data analysis with scRNA-seq data analysis tools in R (Version 3.6.3). Seurat (Version 3.2.0) [17] was mainly used in data integration and downstream analysis. Quality of cells was evaluated based on two metrics; cells with percent.mt ≥ 15% and gene number ≤ 500 were filtered. The datasets were processed using the same quality control parameters. *IntegrateData* function was performed for data integration to eliminate batch effect. Further, unsupervised clustering analyses were performed and the first 20 PCs (principal components) were applied for Uniform Manifold Approximation and Projection (UMAP) analysis. Cell clustering analyses were performed with *FindClusters* function. The resolution parameter was set at 0.5 in this study. Next, cell markers among clusters were identified by *FindAllMarkers* and *FindMarkers* functions. Clusters were then annotated based on the expression of known genes. Cell types were annotated with cell markers and the R package SingleR [18].

### 2.3. Cell-Cell Interaction Event Annotation for Functional Alteration Analysis

Curated receptors, ligands, and the interactions were included in CellPhoneDB (Version 2.1.5) [11], which is a publicly available repository. CellPhoneDB allows in searching for particular ligand/receptor or interrogating single-cell transcriptomics data. Aiming to reveal the cell-cell interaction events (CCEs) among different cell types and compare the difference between healthy liver tissue and HCC liver tissue, cellphoneDB in python (version 3.6.0) was applied and default parameters were used. Further, significant interaction pairs (*p* value < 0.05) were reserved for running the subsequent analyses. According to the annotation in CellPhoneDB, the genes in CCE were separated as ligand and receptor for further studies. The genes annotated as “True” receptor in the interacting pair were accepted as receptors interacted in the CCE. The “False” one was taken as ligand. Ligand-derived cell types were treated as regulatory cells (source cell types), while the receptor-derived cell types were regarded as regulated cells (target cell types). Further, Fisher's exact test was performed to identify CCE-enriched cell types. The CCE with the absolute value of fold change > 0.25 refers to this CCE being differentially interacted and dysregulated in two pathological states [19]. The function of ligands and receptors in CCEs was annotated for enrichment analysis to reveal the alteration of biological processes and pathways in target cells.

### 2.4. Immune Cell Infiltration in a Microenvironment

Transcriptome profiles in TCGA LIHC datasets were analyzed for identification of immune microenvironment changes and prognosis related features in HCC. CIBERSORT [10] was performed with count data to estimate immune infiltrates of tumor samples in TCGA LIHC. There are 22 infiltrated immune cell types predicted. The proportion of infiltrated immune cells in nontumor samples and tumor samples were compared with Wilcoxon's test (two-sided). The univariable cox regression analysis and Kaplan–Meier estimate were applied for the identification of progression (overall survival (OS))-associated immune cell types. The feature with *p* value < 0.05 in univariable cox regression analysis was identified as significantly associated with OS. While for Kaplan-Meier estimate, the patients were divided into two groups by the infiltrated proportion of cells. The number of patients in the group should be greater than 10% of all patients. The features with log-rank *p* value < 0.05 were associated with patients' progression.

### 2.5. CCE-Based Prognosis Signature Construction

To reveal the CCE functions in HCC progression, the CCE genes were used to construct a CCE-based prognosis signature. The univariable cox regression analysis was applied for the identification of progression- (OS-) associated CCE genes. The transcriptome profiles of HCC patients in TCGA HCC dataset were set as a training set (371 samples). The GSE14520 dataset was set as a test set. The genes with *p* value < 0.05 were identified as significance associated with OS. In addition, to establish a prognostic signature, multivariable Cox regression analyses were performed with significant (*p* value < 0.05) progression-related genes. The Akaike Information Criterion (AIC) statistic was used to select a model with function *step* in R package stats. To graphically exhibit the prognostic outcomes, samples was separated into the high-risk group and the low-risk group with the median of the risk score as cutoff, and Kaplan Meier (KM) survival curves were generated. The signature was validated in the test set.

### 2.6. Statistical Analysis and Functional Enrichment Analysis

Genes annotated with different functions were clustered by Gene ontology and KEGG. Functional enrichment analysis was performed with genes by clusterProfiler (version 3.10.1) [20] in R. Enriched terms were kept with adjust *p* value < 0.05. Protein-protein interactions (PPI) were annotated by STRING database (version 11.0) [21]. PPI with combined score ≥ 0.7 were reserved for the next step analysis. Further, Cytoscape (version 3.7.2) [22] was used to construct the immune related gene regulatory network. All the statistical analyses in this study were calculated in R (version 4.0.3) and Python (version 3.7.7). Figures were plotted by the correspondence R package or by ggplot2 (version 3.1.1) in R.

## 3. Results

### 3.1. Tumor Microenvironment Alteration Identified by scRNA-seq Data Analysis

**Figure 1:**
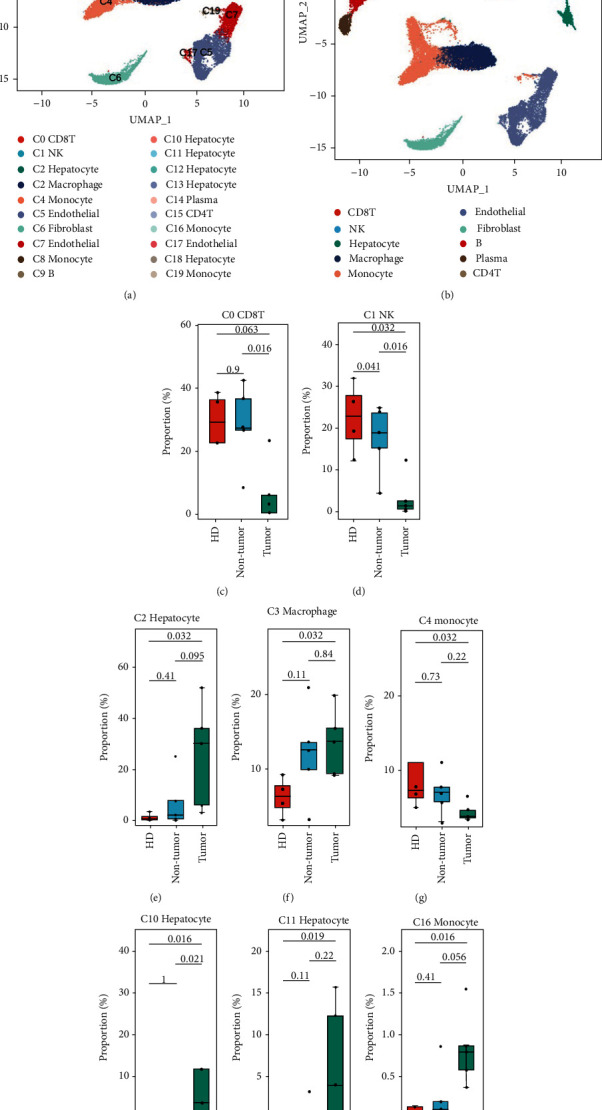
Diverse cell types in the liver microenvironment of HD, NT, and HCC delineated by single-cell RNA-seq analysis. (a) The UMAP plot demonstrates cell clusters in the microenvironment. (b) The UMAP plot demonstrates main cell types in the microenvironment. (c–j) Boxplot of the cell clusters with significant proportion change in the pathological states.

Cells from tumor, paired nontumor liver (NT, paracancerous liver tissue), and healthy donor liver tissue (HD) were divided into 20 clusters by the expression patterns. Single cells could be assigned to distinct cell types using known marker genes (Figures 1(a) and 1(b)). Eight major cell types were noted, including hepatocytes (clusters C2, C10, C11, C12, C13, and C18), endothelial cells (clusters C5, C7, and C17), fibroblasts (cluster C6), and five types of immune cells including T cells (clusters C0 and C15), B cells (clusters C9 and C14), natural killer cells (NK cells) (cluster C1), macrophage (cluster C3), and monocyte (clusters C4, C8, C16, and C19). To compare the proportions of cell clusters of samples in different pathological states, Wilcoxon's test was performed. The clusters including C0, C1, C2, C3, C4, C10, C11, and C16 were found significantly changed between healthy liver (HD) or nontumor liver (NT) and liver tumor tissues (HCC) (Figures 1(c)–1(j)). There is no significant cell proportion change between HD and NT states. However, the hepatocytes (clusters C2, C10, and C11) from HCC tumor samples were found with a higher proportion than in HD or NT states. In addition, the immune cell types including CD8+ T cells (C0), NK cells (C1), and monocytes (C4) were with higher proportions in HD or NT samples. The cell proportion change may be associated with the immunosuppressive microenvironment in HCC.

### 3.2. Infiltrated Immune Cell Proportion Changes Associated with HCC Progression

**Figure 2:**
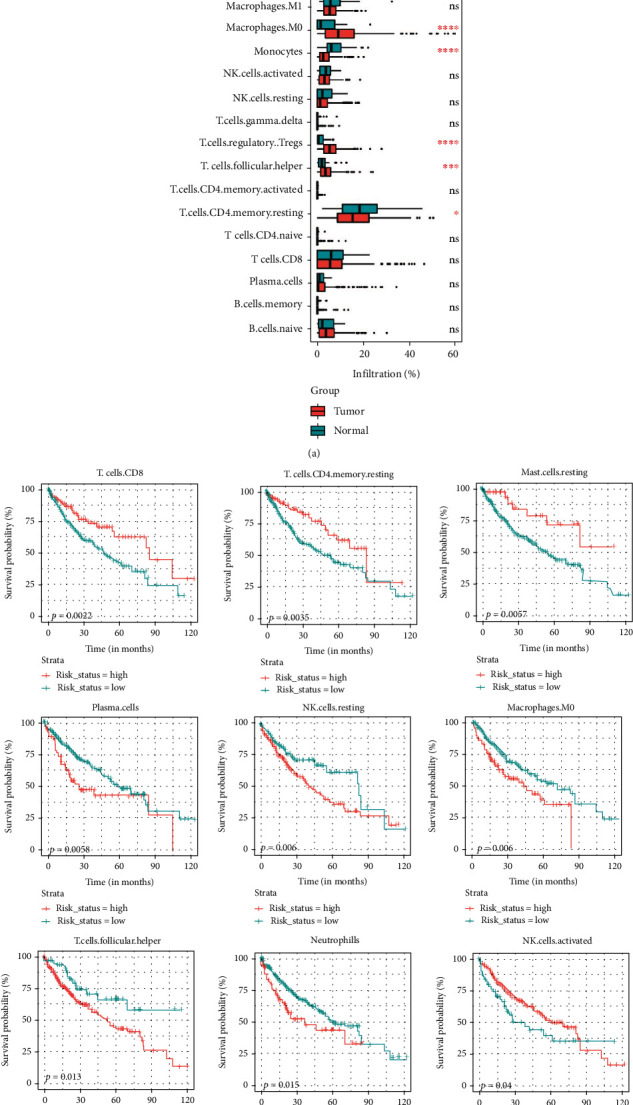
Infiltrated immune cells in HCC associated with patients' prognosis. (a) Boxplot of the infiltrated immune cells shown the significantly change in immune microenvironment. (b) KM-plot of the cells with infiltrated proportions predicted by CIBERSORT.

The infiltrated proportions of immune cells were estimated with the transcriptome profiles of HCC samples in TCGA HCC dataset to reveal the role of proportionally changed immune cells. CIBERSORT was performed to predict the proportion of the 22 immune cell types in HCC and nontumor samples. There were 8 immune cell types with significantly different proportions between tumor samples and nontumor samples (Figure 2(a)). The predicted macrophage M0 was significantly increased in tumor which was consistent with the increasing proportion of macrophage cluster C3. Further, the increasing proportion of macrophage M0 was associated with a poor progression (Figure 2(b)). The monocytes were found with significantly decreased proportions in tumor samples, which was consistent with the main monocyte cluster C4 decreasing in a tumor microenvironment. The predicted CD8+ T cells and two NK cell subtypes were not found significantly changed, while in progression analysis, lower proportion of CD8+ T cells and activated NK cells and higher proportion of resting NK cells were found with significantly lower survival probability (Figure 2(b)). In addition, the neutrophils, activated mast cells, macrophage M2, regulatory T cells (Tregs), follicular help T cells, and resting memory CD4+ T cells demonstrated significantly different proportions. The infiltrated proportions of resting memory CD4+ T cells, follicular help T cells, neutrophils, resting mast cells, and plasma cells were significantly associated with patients' progression. The infiltrated immune cells' proportion changed between NT and HCC, which may also be associated with the progression of patients with HCC.

### 3.3. Dysregulated CCEs in Tumor Cells That Interacted with a Microenvironment

**Figure 3:**
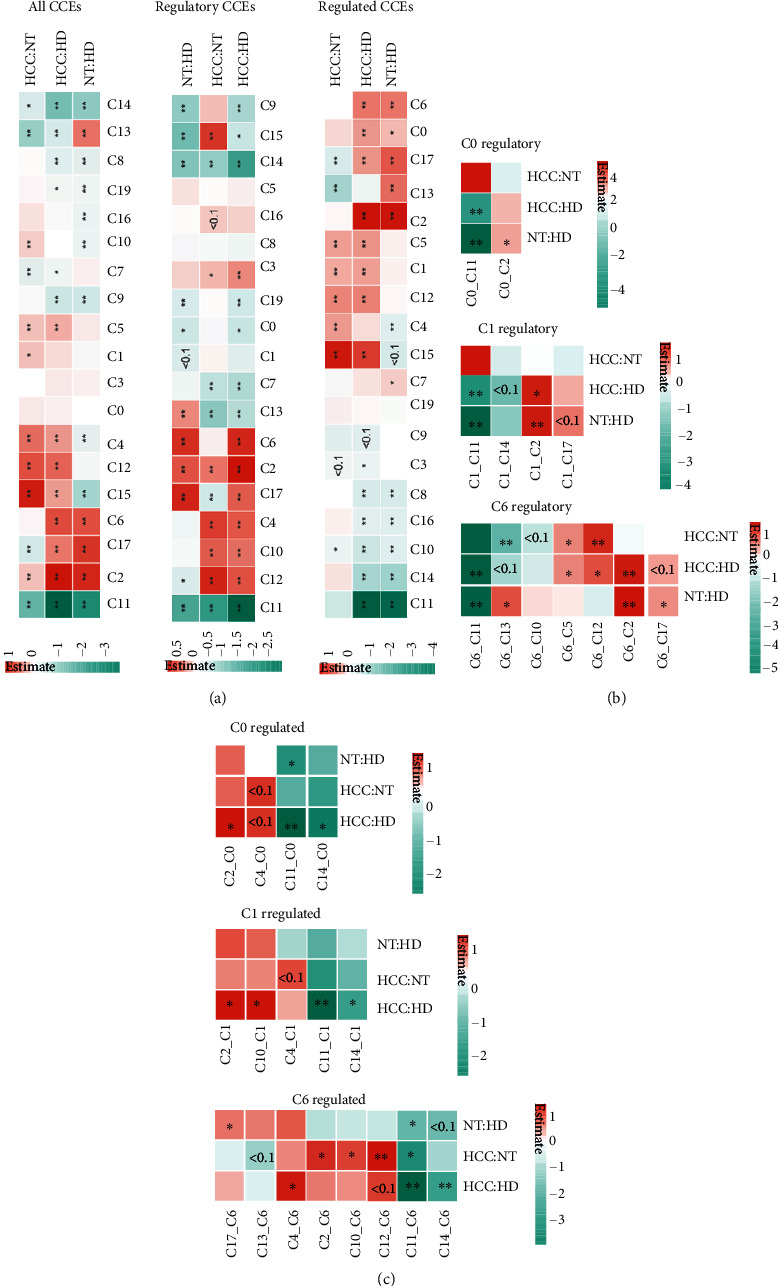
Significantly changed CCE number in microenvironment. (a) The results of Fisher's exact test when calculated the all CCEs, regulatory CCEs, and regulated CCEs of each cell clusters (color bar means the estimate of the odds ratio after log2). (b) The results of Fisher's exact test when calculated the regulatory CCEs of each interacted cell cluster pair. (c) The results of Fisher's exact test when calculating the regulated CCEs of each interacted cell cluster pair.

Cellular interactions in microenvironment were annotated with cellphoneDB to reveal the functional alteration when infiltrated immune cell proportion changed. There are 13223, 10238, and 10226 CCEs identified in healthy donors (HD), paired nontumor tissue (NT), and liver tumor tissue (HCC) separately. Fisher's exact test was performed to check the significant CCE number gain or loss. Cluster C0 and cluster C1 with lower proportion in HCC were found with more CCEs in HCC and NT than in HD when C0 was regulated by the other cell clusters (Figure 3(a)). The number of CCEs interacted in C2_C0, C2_C1, and C10_C1 was significantly increased (Figures 3(b) and 3(c)). There appeared not only clusters with the significantly different number of CCEs between HCC and NT or HD but also some clusters with significantly different number of CCEs between NT and HD including the hepatocyte cluster C2 and the fibroblast cluster C6 and so on (Figure 3). The proportions of fibroblast were not significantly gained or lost between pathological states. However, the number of CCEs between HCC or NT and HD was significantly a gain (Figure 3(a)). The interacted cell pairs with increased CCEs including C6_C2. The interacted cell pair C2_C6 was also significantly increased in CCE number (Figures 3(b) and 3(c)). Further, dysregulated CCEs between states were identified.

**Figure 4:**
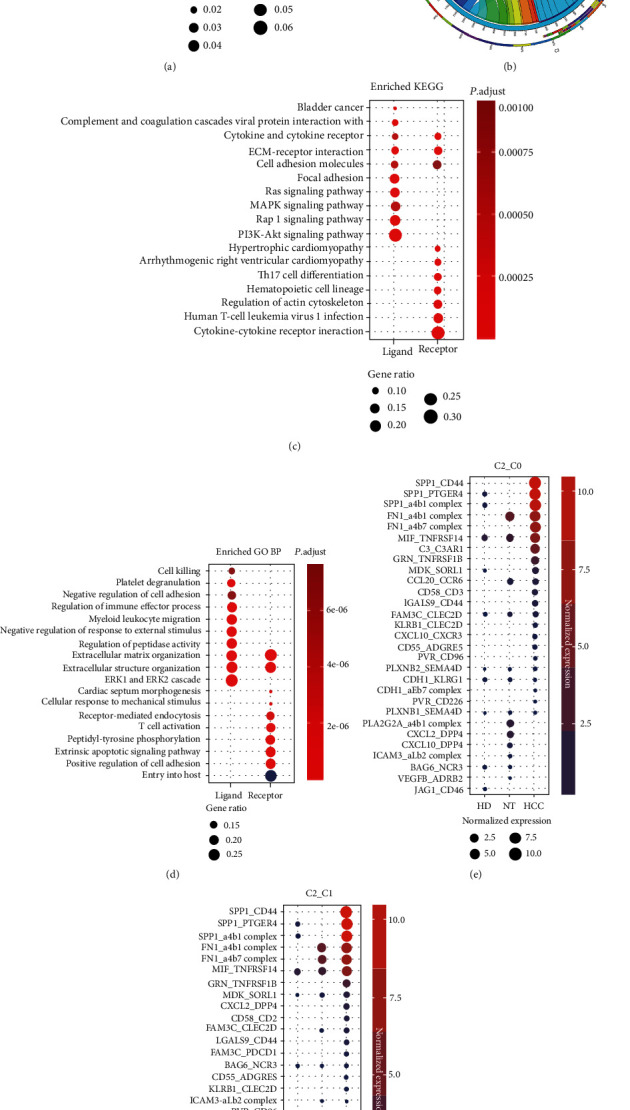
Cell-cell interaction events (CCEs) between hepatocyte (cluster C2) and other cell clusters in the microenvironment. (a) Significantly enriched KEGG pathways of the markers of cluster C2. (b) The circos plot for CCE counts from hepatocyte cluster to other cell clusters in HCC. (c) Significantly enriched KEGG pathways of the ligand and receptor genes of cluster C2 in HCC. (d) Significantly enriched GO BP terms of the ligand and receptor genes of cluster C2 in HCC. (e) Dysregulated CCEs when C2 interacted with C0, C2 as the regulatory cell cluster. (f) Dysregulated CCEs when C2 interacted with C1, C2 as the regulatory cell cluster.

Cluster C2 was the main hepatocyte cluster. Hepatocyte cluster C2 was found with more CCEs in HCC than in NT and HD. The markers of C2 are significantly enriched in KEGG pathways including nonalcoholic fatty liver disease, alcoholic liver disease, and ferroptosis (Figure 4(a)). The hepatocytes from HCC would be malignant cells. Further, C2 interacted with other cell clusters (Figure 4(b)) by CCEs including MIF_TNFRSF14, SPP1_CD44, and VEGFA_NRP1 (Figures 4(e) and 4(f), Figure S1). SPP1 which was reported mainly expressed in malignant cells could act as a driver of tumor evolution [23]. The SPP1-CD44 interacted between malignant cells and T cells may support the key role of SPP1 in tumor ecosystem [23]. When hepatocyte cluster C2 interacts with fibroblast cluster C6, JAG1-NOTCH3 interacts in HD; this is involved in the regulation of cell fate in target fibroblasts. The loss of this regulatory relationship in HCC may contribute to the dysregulation of fibroblast fate [24]. In addition, VEGFA-NRP1 and VEGFA-NRP2 interacting in HCC participate in signaling pathways controlling cell migration [25]. These increasing interactions might play important roles in TME maintenance and tumor progression.

To reveal the functions of ligands and receptors in malignant hepatocytes, function enrichment analysis was performed. In HCC, the ligand genes in C2 are mainly enriched in signaling pathways including PI3K-Akt signaling pathway, Rap1 signaling pathway, Ras signaling pathway, and ECM-receptor interaction (Figure 4(c)). Biological processes including extracellular structure organization, regulation of immune effector, myeloid leukocyte migration, and cell killing are significantly enriched (Figure 4(d)), while the receptor genes in malignant hepatocytes are mainly involved in regulation of actin cytoskeleton, extrinsic apoptotic signaling pathway, and peptidyl-tyrosine phosphorylation. The cellular interactions between malignant cells and microenvironment reveal the potential immune regulation and immune escape in tumor development.

Clusters C10 and C11 are both annotated as hepatocytes, too. The hepatocytes from HCC have significantly higher proportion than NT and HD. The cell markers of cluster C10 and C11 were also enriched in KEGG pathways including chemical carcinogenesis, nonalcoholic fatty liver disease, and drug metabolism (Figure S2, S3). The ligand genes and receptor genes of HCC in C10 and C11 were significantly enriched in the KEGG pathways and GO BP terms including regulation of immune effector process and peptidyl-tyrosine phosphorylation. The CCEs including MIF-TNFRSF14 and SPP1-CD44 also significantly interacted between hepatocyte cluster C10 and other cell clusters. However, the CCEs of C11 were different from C2 and C10. C11 were found with less CCEs in HCC than in HD and NT. The C11 mainly interacted with the other cell clusters including macrophage cluster C3 by MIF_CD74, APP_CD74, and COPA_CD74. The ligand gene MIF is macrophage migration inhibitory factor, which is a proinflammatory cytokine [26] and an oncogene [27]. The receptor gene CD74 may participate in the regulation of antigen presentation for immune response. The interaction of MIF and CD74 was reported to exert proproliferative and antiapoptotic effects in murine hepatocellular carcinoma [28].

### 3.4. Hub Genes in Cell Communication Network Affect Clinical Outcomes

**Figure 5:**
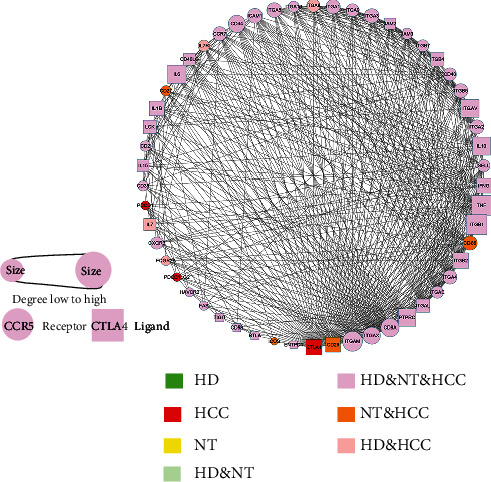
The protein-protein interaction (PPI) network constructed with HCC-specific CCE gene CTLA4 and the related CCE genes in HCC.

In order to clarify the relationship between gene alteration and microenvironment in the liver, 480 receptor and ligand genes were used to construct the integrated interaction network. Pathological state-specific interaction networks were extracted, then. CTLA4 was uniquely identified with a higher degree in the HCC interaction network (Figure 5), while in HCC, CTLA4 interacted with CD86, which may participate negatively in the regulation of T cell activation and diminishment of immune response [29]. ITGB3 and CD80 were uniquely identified in nontumor tissue (NT), then (Figure S4). The proteins encoded by ITGB3 and ITGAV integrated as the aVb3 complex interacting with genes including SPP1, F2 in NT. ITGB3 was reported to play a central role in intracellular communication via extracellular vesicles, which was proposed to be critical for cancer metastasis [30]. CD80 is involved in the costimulatory signal essential for T-lymphocyte activation. The CCE CD28_CD80 that interacted in NT could induce T-cell proliferation and cytokine production. In healthy donors (HD), CXCL8 is uniquely interacted with the Atypical Chemokine Receptor 1 (ACKR1) (Figure S5). CXCL8 is a major mediator of the inflammatory response.

### 3.5. CCE-Based Prognostic Signature Construction

**Figure 6:**
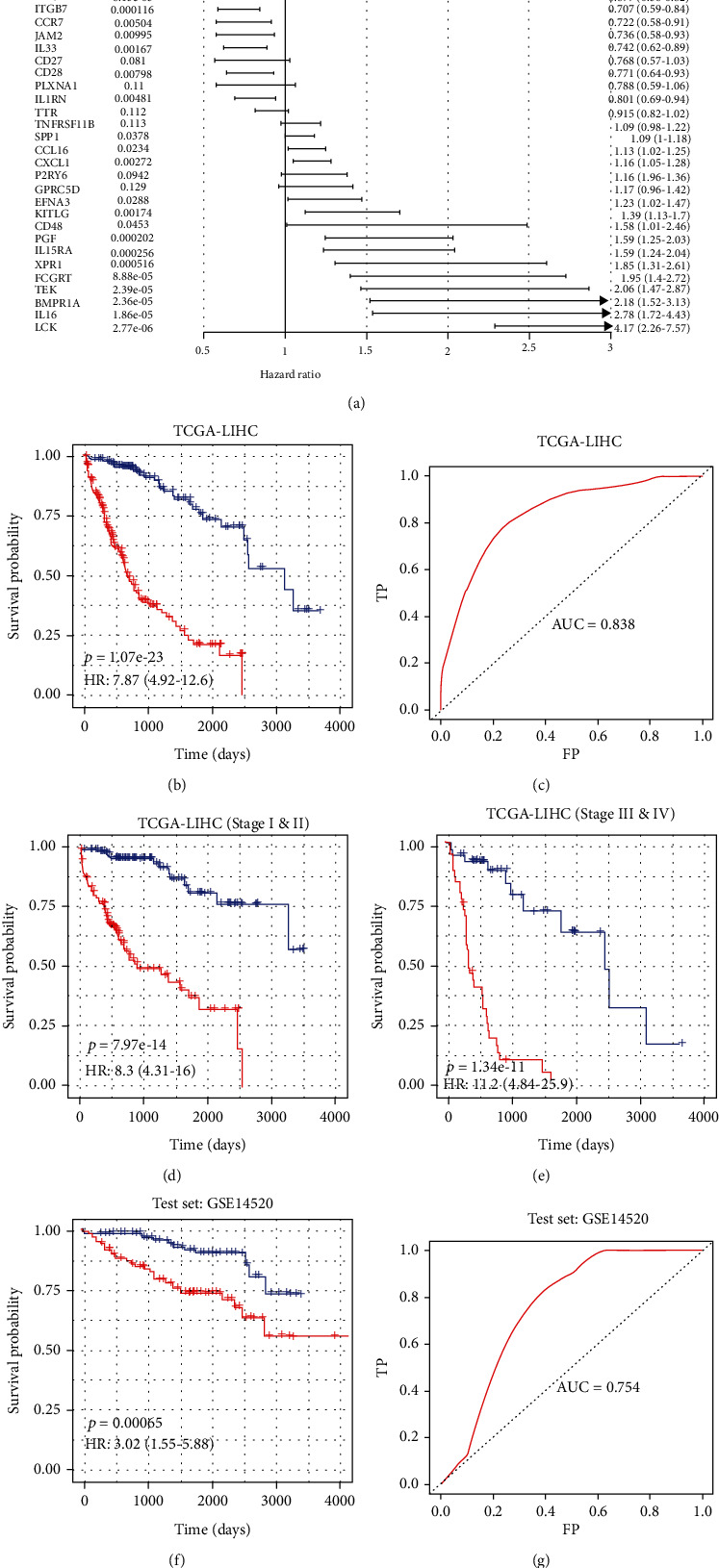
Construction of the prognosis model based on the CCE genes in TME of HCC. (a) The hazard ratio of the genes in the prognosis model. (b) Kaplan-Meier estimates of OS of HCC patients in TCGA as the training datasets based on the 7-gene signature; patients were divided into two risk group according to median risk score. (c) The receiver operating characteristic (ROC) curve for OS survival predictions for the signature in training set. (d) Kaplan-Meier estimates of OS of HCC patients in tumor stage I&II in TCGA based on the signature. (e) Kaplan-Meier estimates of OS of HCC patients in tumor stage III&IV in TCGA based on the signature. (f) Kaplan-Meier estimates of OS of HCC patients in the test datasets based on the signature; patients were divided into two risk group according to median risk score. (g) The receiver operating characteristic (ROC) curve for OS survival predictions for the signature in test set.

To reveal the CCE genes' potential to be candidate markers for HCC, we constructed an immune cell prognostic model for HCC immunoregulatory genes that interacted in cellular interactions. The transcriptomic profiles of the 371 patients with HCC in TCGA were set as the training set, and univariate Cox proportional hazards regression was applied for prognostic marker identification. There were 32 cellular interaction genes selected as the signature by AIC (Figure 6(a)). SPP1 was associated with patients' progression with hazard ratio (HR) > 1. The multivariate Cox risk analysis model was constructed to score the patients' prognostic risk, and patients were divided into the high-risk group and the low-risk group according to the median. The predictive power of the model was robust, and the survival time was significantly shorter in the high-risk group than in the low-risk group (median survival time 660 days (about 1.80 years) vs. 3125 days (about 8.56 years), *p* value < 0.001) (Figure 6(b)). The 1-year AUC value of the ROC curve is 0.838 (Figure 6(c)). Further, the model was confirmed to be a good predictor for progression in patients in both early tumor stage including stage I and II and later tumor stage including III and IV (Figures 6(d) and 6(e)). There was a significant difference between the high-risk and low-risk groups (*p* value < 0.01), and the 1-year AUC is 0.754 (Figures 6(f) and 6(g)).

## 4. Discussion

In this study, we classified and annotated cell types in hepatocellular carcinoma, paracancerous tissues, and healthy liver tissues. CCEs in the microenvironment were annotated and compared. The interaction between malignant cells and other types of cells was analyzed in HCC. Functional enrichment analysis was performed to interpret the biological function alteration of the genes in CCE. The heterogeneity of HCC TME immunoregulation was related to dysregulated CCEs that participate in immunoregulation and immune escape. The genes involved in CCEs were associated with the tumorigenesis and progression of HCC. A CCE-based immune regulatory network was extracted to illustrate the mechanism of TME dysregulation.

The number of CCEs in cell clusters was found significantly gained or lost between different pathological states including NT and HD. However, the cell proportions were only significantly changed in the 8 clusters and between HCC and NT or HD. The possible reasons may be that tumor-promoting inflammation in the liver such as hepatitis virus infection remains a common feature of the pathogenesis of HCC among all etiologies, and these inflammations are often accompanied by extensive immune infiltration, immune microenvironment change, and CCE alteration. Dysregulated CCEs in the surrounding liver tissue of HCC tumor may be involved in the tumorigenesis and development of tumors. ITGB3 and CD80 are unique CCE genes in NT. ITGB3 was reported as a central role in intracellular communication via extracellular vesicles (PMID: 32848136). CD80 is involved in the costimulatory signal essential for T-lymphocyte activation. The CCE CD28_CD80 that interacted in NT could induce T-cell proliferation and cytokine production. If the CCE gene CD80 interacted with CTLA4 in HCC, it will inhibit T cell activation [31]. The unique CCE hub gene CXCL8 in HD is a major mediator of the inflammatory response. In a word, the paracancerous tissue (NT) may be the intermediate state with dysregulated CCEs between HD and HCC.

Dysregulated CCEs including SPP1-CD44, MIF-TNFRSF14, VEGFA-NRP1, and JAG1-NOTCH3 were found interacting between malignant cells and the other cell clusters. SPP1 encoded osteopontin (OPN), the physiological ligand for CD44, acts as an immune checkpoint to suppress T cell activation and confers host tumor immune tolerance in human colon carcinoma that correlated with decreased patient survival [32]. The interacting pair SPP1-CD44 was significantly present in malignant cell clusters (C2, C10) and other cell clusters in this study. The SPP1 functions may be associated with cell proliferation and apoptosis in HCC [33]. Our collaborative previous studies found that OPN is a promoter for HCC progression; it could induce EMT of HCC cells through increasing vimentin stability [34]. The CCE SPP1-CD44 was reported to trigger the polarization of macrophages in HCC, which was validated by an in vitro experiment [35]. VEGFA-NRP1|NRP2, significantly interacting in malignant cells and fibroblasts in HCC, may participate in signaling pathways that control cell migration, clonogenesis, and self-renewal capacities [25, 36]. Dysregulated CCEs participate in the immune microenvironment reshaping and tumor development.

To clarify the relationship between CCEs and tumor progression, we constructed a multigene tumor prognosis prediction model with TCGA HCC expression datasets. SPP1, LCK, and CCR5 were included as predictors for prognosis prediction. LCK is a protooncogene, a member of the Src family of protein tyrosine kinases (PTK), and the protein encoded by it is a key signal molecule for the selection and maturation of developing T cells. The signature was a good predictor both in the test set and patients in TCGA HCC cohort with different tumor stages. The above reports combined with the results in this study reveal the pivotal role of CCEs in TME of HCC.

In conclusion, we have constructed the microenvironmental CCE landscape of HCC. To avoid the immune heterogeneity in different samples, we tried to interpret the mechanism of HCC progression from paired tumor and nontumor tissues. This workflow can be taken as an important single-cell technology analysis strategy for other tumor microenvironment interaction researches.

## Data Availability

The datasets presented in this study can be found in online repositories. The names of the repository/repositories and accession number(s) can be found below: GSE136103, https://www.ncbi.nlm.nih.gov/geo/query/acc.cgi?acc=GSE136103; GSE149614, https://www.ncbi.nlm.nih.gov/geo/query/acc.cgi?acc=GSE149614; and GSE14520, https://www.ncbi.nlm.nih.gov/geo/query/acc.cgi?acc=GSE14520.

## Supplementary Materials

Supplementary Materials Figure S1: the CCEs of cell cluster C2 interacted with other cell clusters. Figure S2: cell-cell interaction events (CCEs) between hepatocyte (cluster C10) and other cell clusters in the microenvironment. (A) Significantly enriched KEGG pathways of the markers of cluster C10; (B) the circos plot for CCE counts from hepatocyte cluster to other cell clusters in HCC; (C) significantly enriched KEGG pathways of the ligand and receptor genes of cluster C10 in HCC; (D) significantly enriched GO BP terms of the ligand and receptor genes of cluster C10 in HCC; (E) the CCEs of cell cluster C10 interacted with other cell clusters. Figure S3: cell-cell interaction events (CCEs) between hepatocyte (cluster C11) and other cell clusters in the microenvironment. (A) Significantly enriched KEGG pathways of the markers of cluster C11; (B) the circos plot for CCE counts from hepatocyte cluster to other cell clusters in HCC; (C) significantly enriched KEGG pathways of the ligand and receptor genes of cluster C11 in HCC; (D) significantly enriched GO BP terms of the ligand and receptor genes of cluster C11 in HCC; (E) the CCEs of cell cluster C11 interacted with other cell clusters. Figure S4: the protein-protein interaction (PPI) network constructed with NT specific CCE gene ITGB3 and the related CCE genes in NT. Figure S5: the protein-protein interaction (PPI) network constructed with HD specific CCE gene CXCL8 and the related CCE genes in HD.
