# Supplementary material for: Single-Cell RNA-seq Analysis Reveals Dysregulated Cell-Cell Interactions in a Tumor Microenvironment Related to HCC Development
Source: Dis Markers. 2022 May 18;2022:4971621. doi: 10.1155/2022/4971621 (PMC9132707; doi:10.1155/2022/4971621)
Supplement: Supplementary Materials — Figure S1: the CCEs of cell cluster C2 interacted with other cell clusters. Figure S2: cell-cell interaction events (CCEs) between hepatocyte (cluster C10) and other cell clusters in the microenvironment. (A) Significantly enriched KEGG pathways of the markers of cluster C10; (B) the circos plot for CCE counts from hepatocyte cluster to other cell clusters in HCC; (C) significantly enriched KEGG pathways of the ligand and receptor genes of cluster C10 in HCC; (D) significantly enriched GO BP terms of the ligand and receptor genes of cluster C10 in HCC; (E) the CCEs of cell cluster C10 interacted with other cell clusters. Figure S3: cell-cell interaction events (CCEs) between hepatocyte (cluster C11) and other cell clusters in the microenvironment. (A) Significantly enriched KEGG pathways of the markers of cluster C11; (B) the circos plot for CCE counts from hepatocyte cluster to other cell clusters in HCC; (C) significantly enriched KEGG pathways of the ligand and receptor genes of cluster C11 in HCC; (D) significantly enriched GO BP terms of the ligand and receptor genes of cluster C11 in HCC; (E) the CCEs of cell cluster C11 interacted with other cell clusters. Figure S4: the protein-protein interaction (PPI) network constructed with NT specific CCE gene ITGB3 and the related CCE genes in NT. Figure S5: the protein-protein interaction (PPI) network constructed with HD specific CCE gene CXCL8 and the related CCE genes in HD. [file 4971621.f1.pdf]

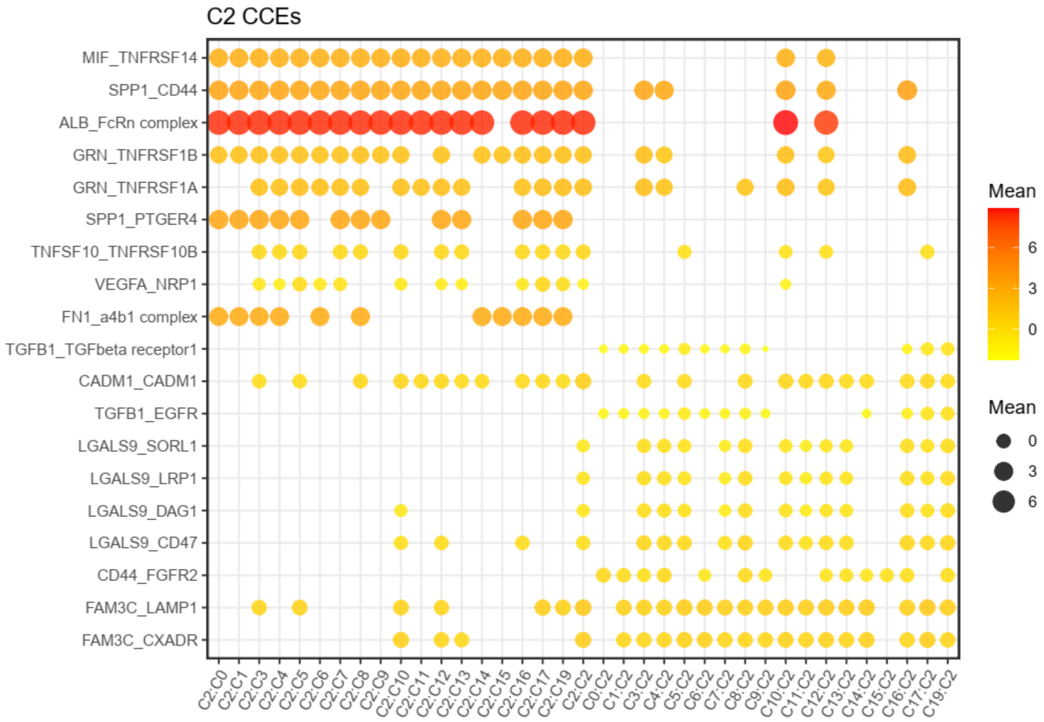

Figure S1. The CCEs of cell cluster C2 interacted with other cell clusters.

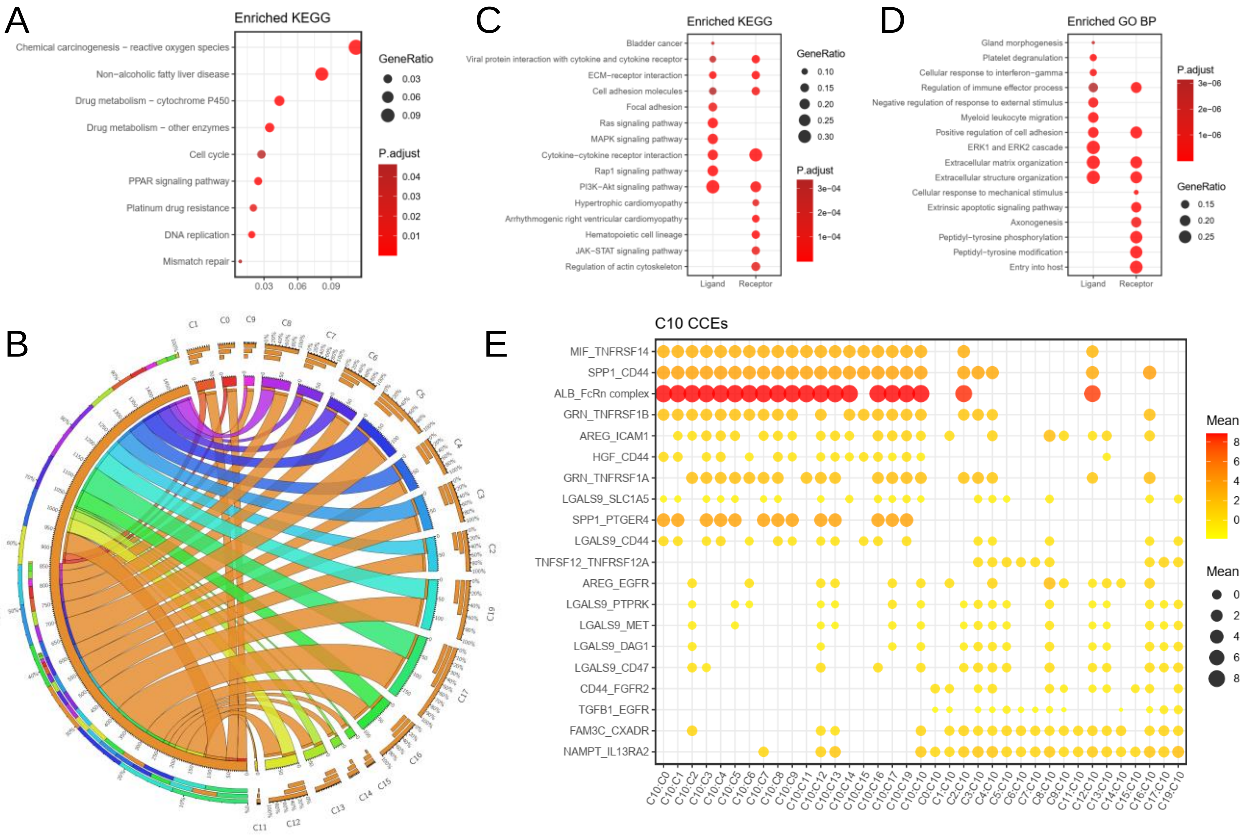

Figure S2. Cell-cell interaction events (CCEs) between hepatocyte (cluster C10) and other cell clusters in the microenvironment.

A) Significantly enriched KEGG pathways of the markers of cluster C10;

B) The circos plot for CCE counts from hepatocyte cluster to other cell clusters in HCC;

C) Significantly enriched KEGG pathways of the ligand and receptor genes of cluster C10 in HCC;

D) Significantly enriched GO BP terms of the ligand and receptor genes of cluster C10 in HCC;

E) The CCEs of cell cluster C10 interacted with other cell clusters.

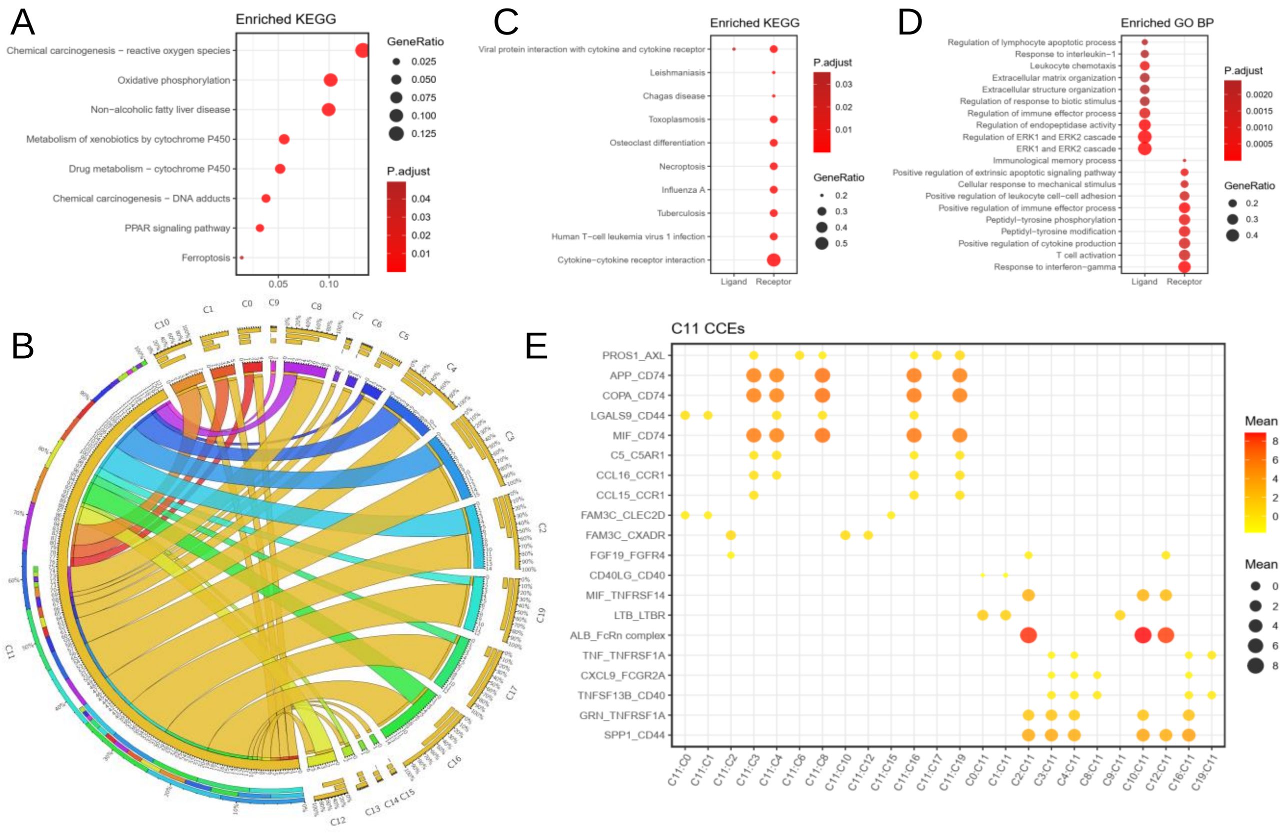

Figure S3. Cell-cell interaction events (CCEs) between hepatocyte (cluster C11) and other cell clusters in the microenvironment.

A) Significantly enriched KEGG pathways of the markers of cluster C11;

B) The circos plot for CCE counts from hepatocyte cluster to other cell clusters in HCC;

C) Significantly enriched KEGG pathways of the ligand and receptor genes of cluster C11 in HCC;

D) Significantly enriched GO BP terms of the ligand and receptor genes of cluster C11 in HCC;

E) The CCEs of cell cluster C11 interacted with other cell clusters.

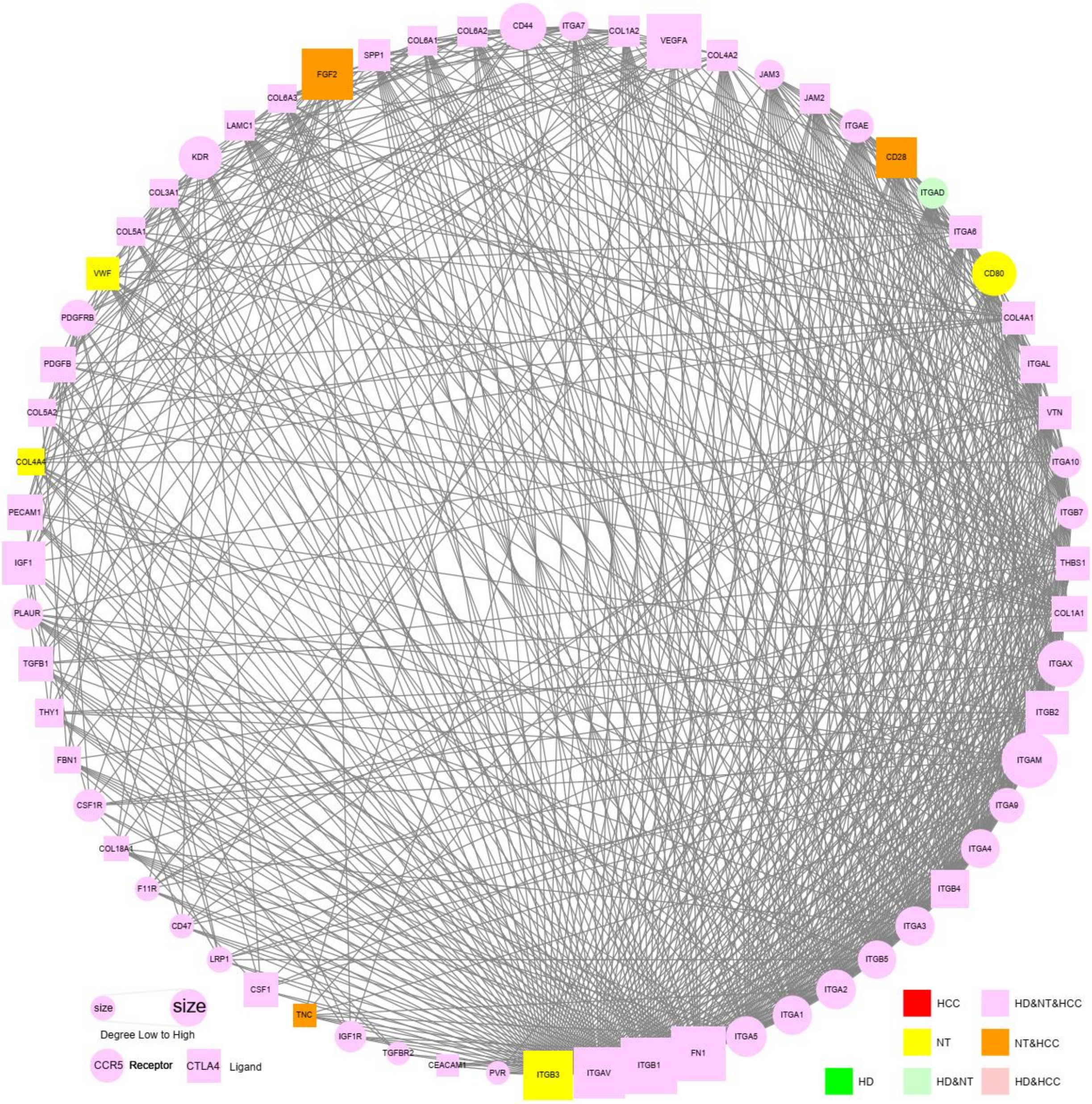

Figure S4. The protein-protein interaction (PPI) network constructed with NT specific CCE gene ITGB3 and the related CCE genes in NT.

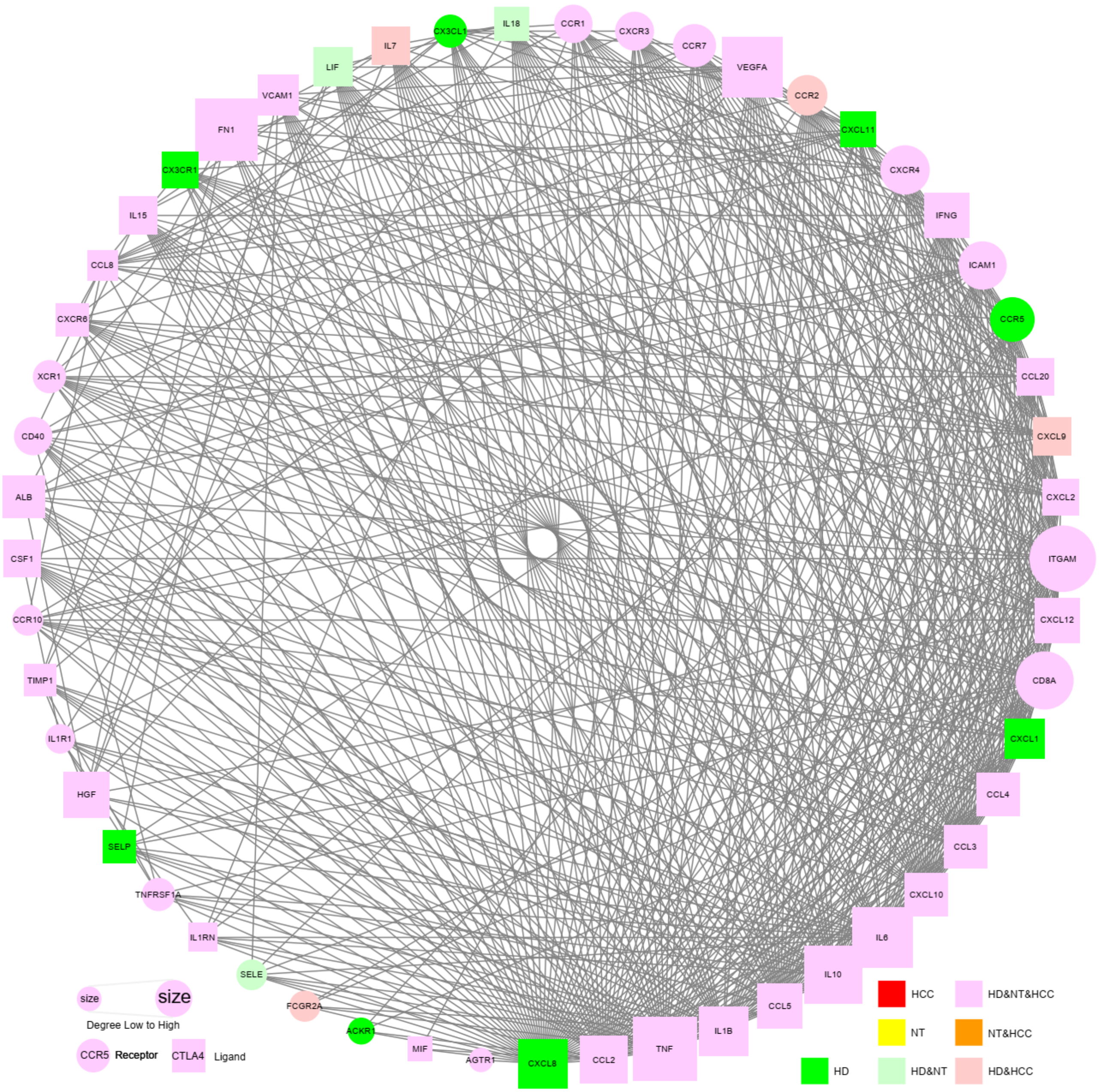

Figure S5. The protein-protein interaction (PPI) network constructed with HD specific CCE gene CXCL8 and the related CCE genes in HD.
